# TonEBP/NFAT5 haploinsufficiency attenuates hippocampal inflammation in high-fat diet/streptozotocin-induced diabetic mice

**DOI:** 10.1038/s41598-017-08319-w

**Published:** 2017-08-10

**Authors:** Jong Youl Lee, Eun Ae Jeong, Kyung Eun Kim, Chin-ok Yi, Zhen Jin, Jung Eun Lee, Dong Hoon Lee, Hyun Joon Kim, Sang Soo Kang, Gyeong Jae Cho, Wan Sung Choi, Soo Youn Choi, H. Moo Kwon, Gu Seob Roh

**Affiliations:** 1 Department of Anatomy and Convergence Medical Science, Institute of Health Sciences, Gyeongsang National University School of Medicine, Jinju, Gyeongnam Republic of Korea; 2 Bio Anti-aging Medical Research Center, Gyeongsang National University School of Medicine, Jinju, Gyeongnam Republic of Korea; 3 Department of Thoracic and Cardiovascular Surgery, Institute of Health Sciences, Gyeongsang National University School of Medicine, Jinju, Gyeongnam, Republic of Korea; 4 School of Nano-Biotechnology and Chemical Engineering, Ulsan National Institute of Science and Technology, Ulsan, Republic of Korea

## Abstract

Recent studies have shown that overexpression of tonicity-responsive enhancer binding protein (TonEBP) is associated with many inflammatory diseases, including diabetes mellitus, which causes neuroinflammation in the hippocampus as well as hepatic steatosis. However, the exact mechanism in diabetic neuroinflammation is unknown. We report that haploinsufficiency of TonEBP inhibits hepatic and hippocampal high-mobility group box-1 (HMGB1) expression in diabetic mice. Here, mice were fed a high-fat diet (HFD) for 16 weeks and received an intraperitoneal injection of 100 mg/kg streptozotocin (STZ) and followed by continued HFD feeding for an additional 4 weeks to induce hyperglycemia and hepatic steatosis. Compared with wild-type diabetic mice, diabetic TonEBP^+/−^ mice showed decreased body weight, fat mass, hepatic steatosis, and macrophage infiltration. We also found that adipogenesis and HMGB1 expression in the liver and hippocampus were lower in diabetic TonEBP^+/−^ mice compared with the wild type. Furthermore, iba-1 immunoreactivity in the hippocampus was decreased in diabetic TonEBP^+/−^ mice compared with that in the wild type. Our findings suggest that TonEBP haploinsufficiency suppresses diabetes-associated hepatic steatosis and neuroinflammation.

## Introduction

Diabetes mellitus (DM) is a common chronic inflammatory disease that causes many complications, such as myocardial infarction, retinopathy, and nephropathy^1, 2^, and is considered an important risk factor for mild cognitive impairment and Alzheimer’s disease^3^. Diabetes-related obesity leads to the recruitment of large numbers of macrophages and T cells to excess adipose tissue, producing proinflammatory cytokines, as well as advanced glycation end-products, and resulting in vascular damage^4, 5^. This low-grade chronic inflammation is an important risk factor for systemic insulin resistance and neuroinflammation^6, 7^. There is evidence that high-fat diet (HFD)-induced chronic peripheral inflammation aggravates neuroinflammation and leads to memory impairment^8^. Thus, the inflammatory state may play an important role in the progression of diabetes-induced cognitive changes.

Memory impairment in aged rats with diabetes is exacerbated by inhibiting the signaling of the transcription factor nuclear factor-kappa B (NF-κB) in the hippocampus^9^. However, downregulation of another member of the Rel family of transcription factors, the tonicity-responsive enhancer binding protein (TonEBP, also known as nuclear factor of activated T cells 5)^10^, prevents neuronal death in animal models of streptozotocin (STZ)-induced diabetic retinopathy^11, 12^. TonEBP is abundantly expressed in the liver, heart, brain, and kidney, as well as in activated T cells^13–15^, and is reduced under hypoosmotic conditions^16^. TonEBP mediates the release of inflammatory cytokines in macrophages and the induction of cyclooxygenase 2 in kidneys in response to activation of Toll-like receptor (TLR) 4 under hypertonic conditions^17^. We previously showed that in the brain, TonEBP has an inflammatory role in the pathology of seizures^18^. However, the role of TonEBP in modulating diabetic-mediated pathology has not been studied. Therefore, we examined the diabetic phenotypes and neuroinflammation in mice with TonEBP haploinsufficiency and HFD/STZ-induced diabetes. We identified TonEBP as a critical regulator in both hepatic steatosis and neuroinflammation in diabetes and showed that reducing TonEBP expression protects against diabetes-induced complications.

## Results

### Body weight and fat mass gains are attenuated in TonEBP^+/−^ mice with DM

**Figure 1:**
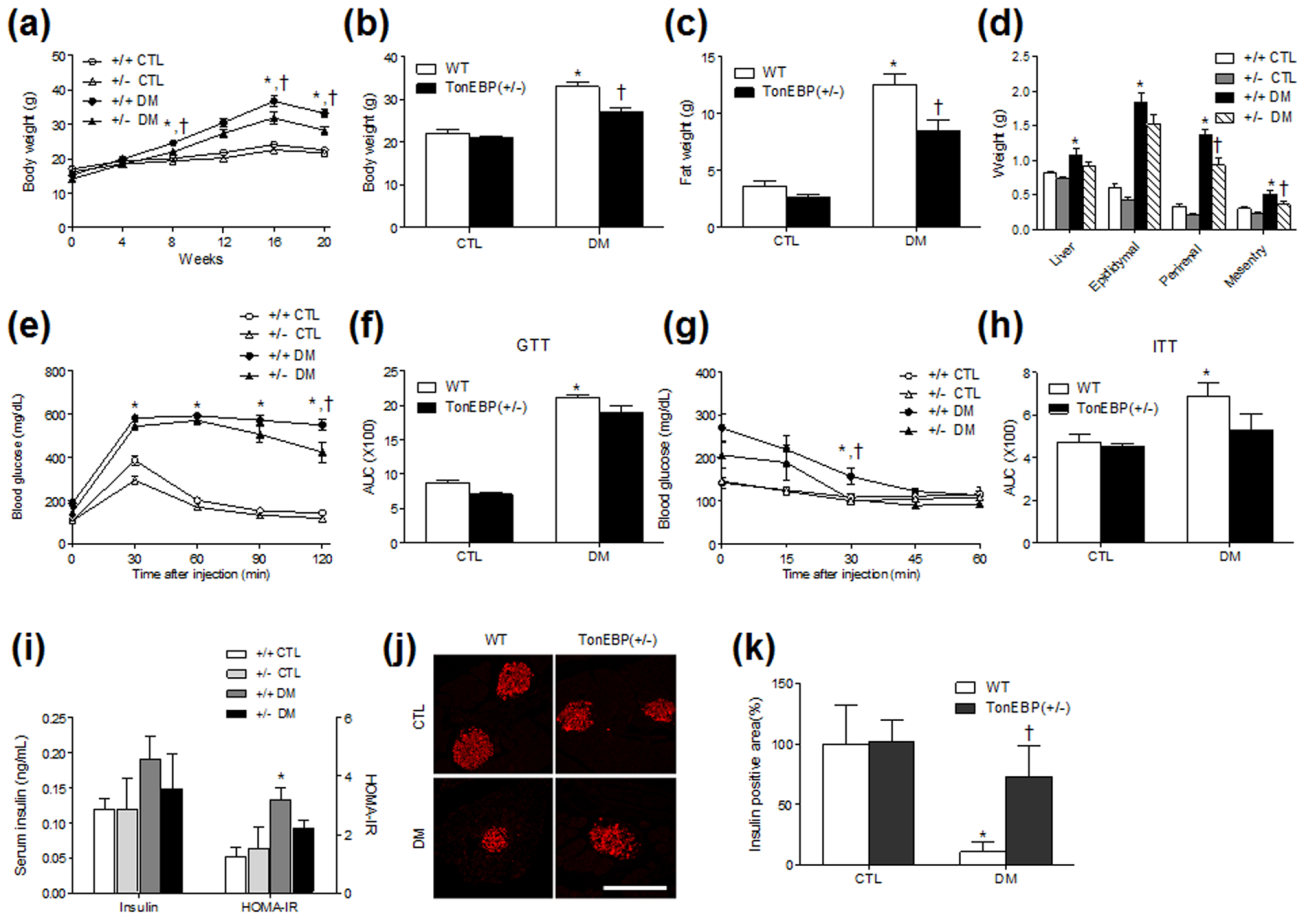
Changes of body weight and metabolic characteristics in HFD/STZ-treated TonEBP^+/−^ mice. (**a**) Body weights of mice for 20 weeks. Body weight (**b**), fat mass (**c**), body fats (**d**), GTT (**e**), areas under the curve (AUC) of GTT (**f**), ITT (**g**), and AUC of ITT (**h**) of WT and TonEBP^+/−^ mice with or without HFD/STZ treatment were compared. (**i**) Serum insulin was measured by an ELISA kit. HOMR-IR was calculated from fasting serum glucose and insulin. Data (n = 10 mice per group) are shown as the mean ± SEM. (**j**) Representative images showing immunofluorescence of insulin (red) in pancreatic sections. Scale bar = 50 μm. (**k**) Percentage areas of insulin-positive cells. Data are shown as the mean ± SEM. *P < 0.05 *vs*. control (CTL) normal diet-fed mice; ^†^ *P* < 0.05 *vs*. DM WT mice.

The effects of HFD/STZ treatment on body weight and fat mass were assessed in mice with TonEBP haploinsufficiency (TonEBP^+/−^) and in wild-type (WT) mice. After 8 weeks on HFD, the body weights of mice with either genotype were significantly increased compared with those in mice fed a normal diet (*P* < 0.05), and the body weights of mice fed an HFD were slightly reduced after an STZ injection at 16 weeks (Fig. 1a). Although the total food intakes were similar (Supplementary Fig. 1a,b), we found that TonEBP^+/−^ mice with DM had lower body weights than WT mice with DM (Fig. 1b). In accordance with changes in body weights, the weights of fat mass, and of perirenal and mesentery fats, from TonEBP^+/−^ mice with DM were significantly reduced compared with those from WT mice with DM (*P* < 0.05) (Fig. 1c,d).

### TonEBP haploinsufficiency attenuates insulin resistance in mice with DM

To determine if TonEBP haploinsufficiency alters the diabetic phenotypes in HFD/STZ-treated mice, we measured the fasting blood glucose and serum insulin levels and responses to the glucose tolerance test (GTT) and the insulin tolerance test (ITT). Animals fed an HFD for 16 weeks showed increased fasting blood glucose levels compared with those fed a normal diet. However, there was an increase in blood glucose levels at 20 weeks in WT and TonEBP^+/−^ mice with normal diets (Supplementary Fig. 2a). This result indicated that some stressful tests (two independent GTT and ITT at 19 weeks) may have caused temporary increases of blood glucose. In contrast to blood glucose levels, we found that there was a significant difference in serum glucose levels between the control (CTL) and DM mice (Supplementary Fig. 2b). GTT and ITT analyses showed that insulin resistance was induced by HFD/STZ treatment (Fig. 1e–h). However, there were no significant changes in serum insulin levels between CTL and DM mice (Fig. 1i). To confirm the effects of TonEBP haploinsufficiency on insulin resistance and β-cell function, we determined the homeostatic model assessment-insulin resistance (HOMA-IR). HOMA-IR was increased in diabetic WT mice compared with those in WT mice with normal diets (Fig. 1i). However, there was slightly reduction of HOMA-IR in diabetic TonEBP^+/−^ mice than in diabetic WT mice. We next examined the effect of HFD/STZ treatment on pancreatic insulin activity in WT and TonEBP^+/−^ mice by immunostaining with an antibody against insulin (Fig. 1j). Compared with normal diet-fed controls, mice with DM exhibited decreased percentages of insulin-positive areas (Fig. 1k). Interestingly, the percentage of insulin-positive areas was lower in WT mice with DM than in TonEBP^+/−^ mice with DM. To differentiate type 1 DM from type 2 DM, immunohistochemical localization of C-peptide in pancreatic sections were evaluated (Supplementary Fig. 2c). The diabetic WT and TonEBP^+/−^ mice exhibited less immunoreactivity of C-peptide compared with normal diet-fed mice (Supplementary Fig. 2d). These findings indicated that HFD/STZ caused β-cell dysfunction and loss of β-cells resulting in decreased insulin secretion.

### TonEBP haploinsufficiency reduces hepatic steatosis and adipogenesis in mice with DM

**Figure 2:**
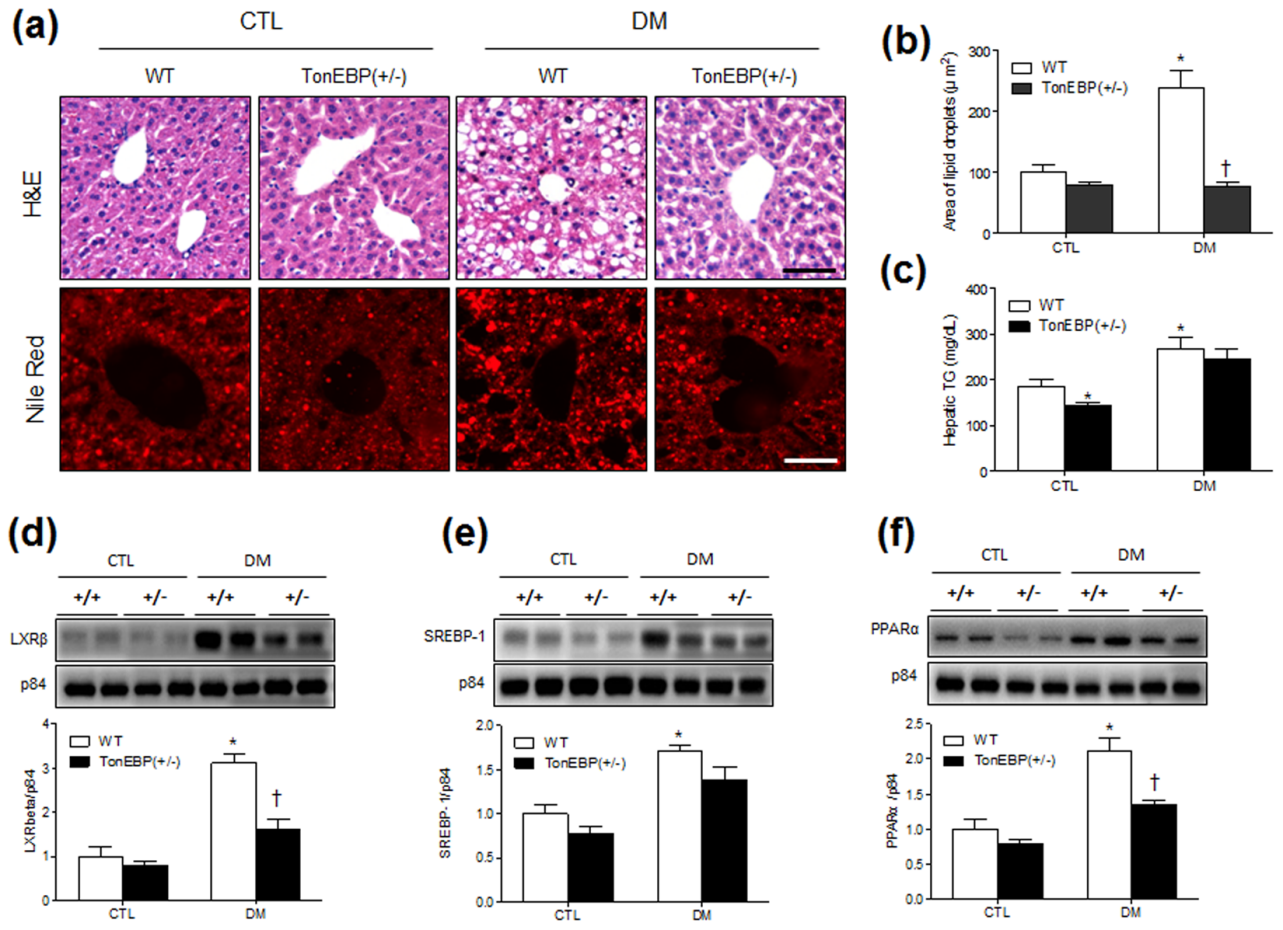
Effects of TonEBP haploinsufficiency on hepatic steatosis in HFD/STZ-treated mice. (**a**) Representative micrographs of H&E and Nile red staining; scale bar = 50 μm. (**b**) Percentages of Nile red-positive areas. (**c**) Concentrations of hepatic TG (n = 7–8 mice per group). Western blotting and quantification of nuclear LXRβ (**d**), SREBP-1 (**e**), and PPARα (**f**) in the livers. The mean values were obtained from three separate experiments (n = 6 mice per group). Cropped blots are displayed here and full-length blots are included in the Supplementary Information. Data are presented as the mean ± SEM. *P < 0.05 *vs*. control (CTL) normal diet-fed mice; ^†^ *P* < 0.05 *vs*. DM WT mice.

We next performed histological examinations of liver sections and western blot analyses. Hematoxylin and eosin (H&E) and Nile red staining showed that hepatic steatosis in WT and TonEBP^+/−^ mice was increased by HFD/STZ (Fig. 2a). However, the areas (from Nile red-stained sections) containing hepatic lipid droplets were reduced in TonEBP^+/−^ mice with DM compared with those in diabetic WT mice (Fig. 2b). Accordingly, we found that hepatic triglyceride (TG) concentrations, which were higher in diabetic mice, were lower in mice with TonEBP haploinsufficiency (Fig. 2c). Hepatic enzymes were also higher in HFD/STZ-treated mice than in those fed a normal diet and were significantly decreased in TonEBP^+/−^ mice with DM compared with the levels in WT mice with DM (Supplementary Fig. 3a).

The synthesis of hepatic TG is transcriptionally regulated by the liver X receptor β (LXRβ), sterol regulatory element-binding protein 1 (SREBP-1), and peroxisome proliferator-activated receptor α (PPARα)^19^. We found that HFD/STZ treatment increased the hepatic expression of these factors in WT and TonEBP^+/−^ mice, while the increases in LXRβ and PPARα were significantly attenuated by TonEBP haploinsufficiency (Fig. 2d–f). In addition, we found that serum total cholesterol levels were higher in mice with DM than in normal diet-fed mice (Supplementary Fig. 3b).

### HFD/STZ-induced TonEBP is reduced in the livers of TonEBP^+/−^ mice

**Figure 3:**
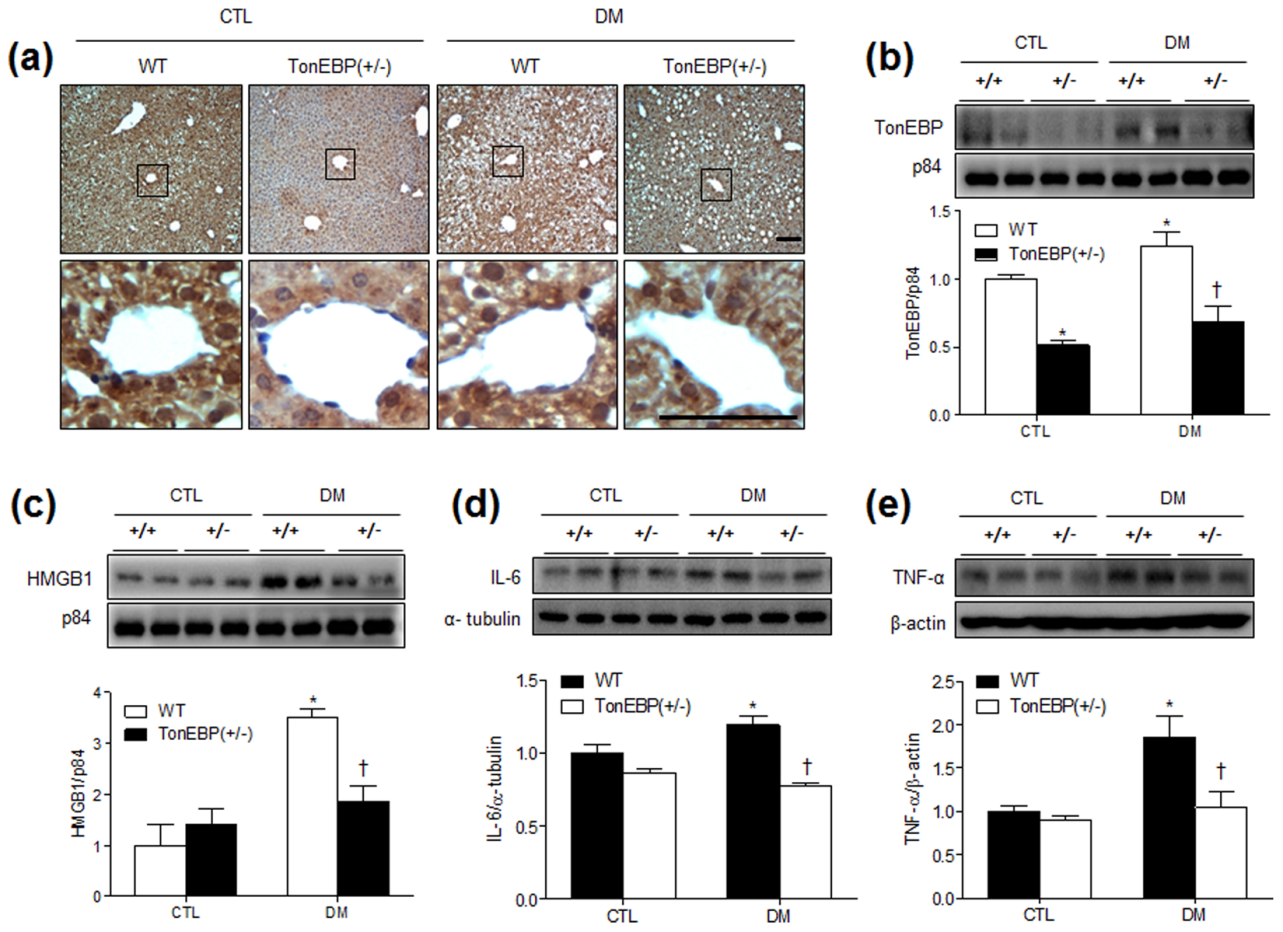
Hepatic TonEBP, HMGB1, IL-6, and TNF-α expression in HFD/STZ-treated TonEBP^+/−^ mice. (**a**) Representative micrographs of TonEBP-immunostained liver sections; scale bar = 50 μm. Western blots and protein quantification of hepatic TonEBP (**b**), HMGB1 (**c**), IL-6 (**d**), and TNF-α (**e**). The mean values were obtained from at least three independent experiment (n = 6 mice per group). Cropped blots are displayed here and full-length blots are included in the Supplementary Information. Data are presented as the mean ± SEM. *P < 0.05 *vs*. control (CTL) normal diet-fed mice; ^†^ *P* < 0.05 *vs*. DM WT mice.

A previous study showed that TonEBP is also expressed in the liver^20^. We examined the expression levels and localization of TonEBP in the liver after HFD/STZ treatment by immunohistochemical staining and western blot analysis. We found that TonEBP is weakly expressed in the nuclei of hepatocytes in TonEBP^+/−^ mice compared with that in WT mice and highly expressed in the nuclei after HFD/STZ treatment (Fig. 3a). This was confirmed quantitatively by western blotting using nuclear fractions of livers from WT and TonEBP^+/−^ mice (Fig. 3b). The expression level of TonEBP from nuclear fractions was significantly increased only in HFD/STZ-treated WT mice.

TonEBP is involved in controlling inflammation in response to TLR activation^21, 22^, and TLR4 is known to mediate high-mobility group box-1 (HMGB1) in association with nonalcoholic fatty liver disease^23^. Thus, we examined whether nuclear HMGB1 expression is associated with HFD/STZ-induced hepatic steatosis. Western blot analysis showed that nuclear protein levels of HMGB1 were decreased in TonEBP^+/−^ mice with DM compared with that in WT mice with DM (Fig. 3c). The levels of hepatic interleukin-6 (IL-6) and tumor necrosis factor- α (TNF-α) expression were also significantly higher in diabetic WT mice compared to normal diet-fed WT mice (Fig. 3d,e). Consistent with the HMGB1 findings, two proinflammatory cytokines were significantly decreased in diabetic TonEBP^+/−^ mice compared with that in diabetic WT mice. These findings indicated that TonEBP haploinsufficiency attenuated HFD/STZ-induced hepatic inflammation. In addition, we found that nuclear NF-κB p65 expression was higher in diabetic than in normal diet-fed WT mice; however, there was no change with respect to TonEBP haploinsufficiency (Supplementary Fig. 4a).

### Macrophage infiltration is reduced in adipose tissues of TonEBP^+/−^ mice

The activation of TLR in macrophages requires the induction of TonEBP^17^. To evaluate the effects of TonEBP haploinsufficiency on macrophage infiltration in HFD/STZ-treated mice, we performed immunohistochemistry for CD68 in adipose tissue sections. We found that the size of adipocytes and CD68 immunoreactivity were higher in HFD/STZ-treated than in normal diet-fed WT mice; however, CD68 immunoreactivity of macrophages, induced by HFD/STZ, was reduced in TonEBP^+/−^ mice (Supplementary Fig. 5a–c). In accordance with hepatic IL-6 expression, we also found that the level of IL-6 expression in adipose tissues was decreased in diabetic TonEBP^+/−^ mice compared with that in diabetic WT mice (Supplementary Fig. 5d). However, the HFD/STZ-induced TNF-α expression in WT mice was slightly reduced in diabetic TonEBP^+/−^ mice (Supplementary Fig. 5e).

### HFD/STZ increases TonEBP expression in hippocampal neurons

**Figure 4:**
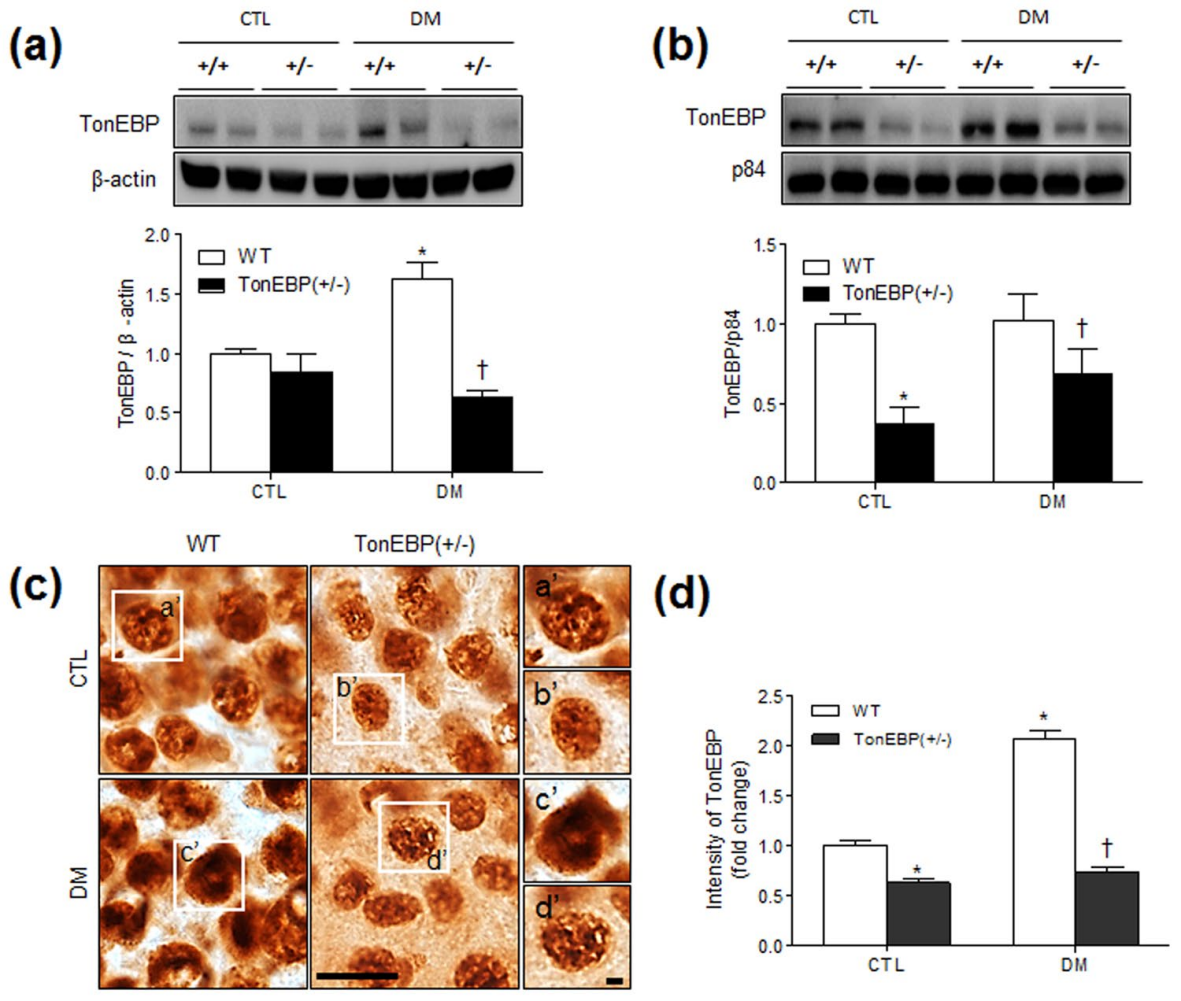
Hippocampal TonEBP expression in HFD/STZ-treated TonEBP^+/−^ mice. Western blots and protein quantification of total (**a**) and nuclear (**b**) TonEBP in the hippocampus. The mean values were obtained from three separate experiments (n = 6 mice per group). Cropped blots are displayed here and full-length blots are included in the Supplementary Information. Data are presented as the means ± SEM. *P < 0.05 *vs*. control (CTL) normal diet-fed mice; ^†^ *P* < 0.05 *vs*. DM WT mice. (**c**) Representative micrographs of TonEBP immunoreactivity in the hippocampus. TonEBP labeling resembles fine sand grains. The boxed areas of a’, b’, c’, and d’ are magnified on the right. Scale bar = 25 μm (inset, 5 μm). (**d**) The intensity of TonEBP immunoreactivity in the hippocampus was measured and presented as the fold change. Data are presented as the mean ± SEM. *P < 0.05 *vs*. control (CTL) normal diet-fed mice; ^†^ *P* < 0.05 *vs*. DM WT mice.

We next examined the effect of DM on the hippocampal expression of TonEBP. To analyze TonEBP expression quantitatively, we performed western blotting using hippocampal total lysates and nuclear fractions from WT and TonEBP^+/−^ mice (Fig. 4a,b). TonEBP from total lysates was slightly decreased in TonEBP^+/−^ mice (*P* = 0.37); however, the nuclear expression level was dramatically decreased in TonEBP^+/−^ mice compared with that in WT mice with normal diets (*P* = 0.007) (Fig. 4b). In particular, we found that TonEBP expression levels from total lysates were increased by HFD/STZ in WT mice compared with that in WT mice with normal diet (Fig. 4a). TonEBP expression levels from both total lysates and nuclear fractions were dramatically decreased in diabetic TonEBP^+/−^ mice compared with that in diabetic WT mice. Accordingly, immunohistochemical staining showed that nuclear TonEBP in hippocampal neurons was increased in WT mice with DM, whereas the expression was relatively weak in TonEBP^+/−^ DM mice (Fig. 4c,d). Double immunofluorescence staining showed that NeuN-positive neurons from WT and TonEBP^+/−^ mice showed strong TonEBP nuclear staining (Supplementary Fig. 6).

### HFD/STZ-induced HMGB1 is abolished in the hippocampi of TonEBP^+/−^ mice

**Figure 5:**
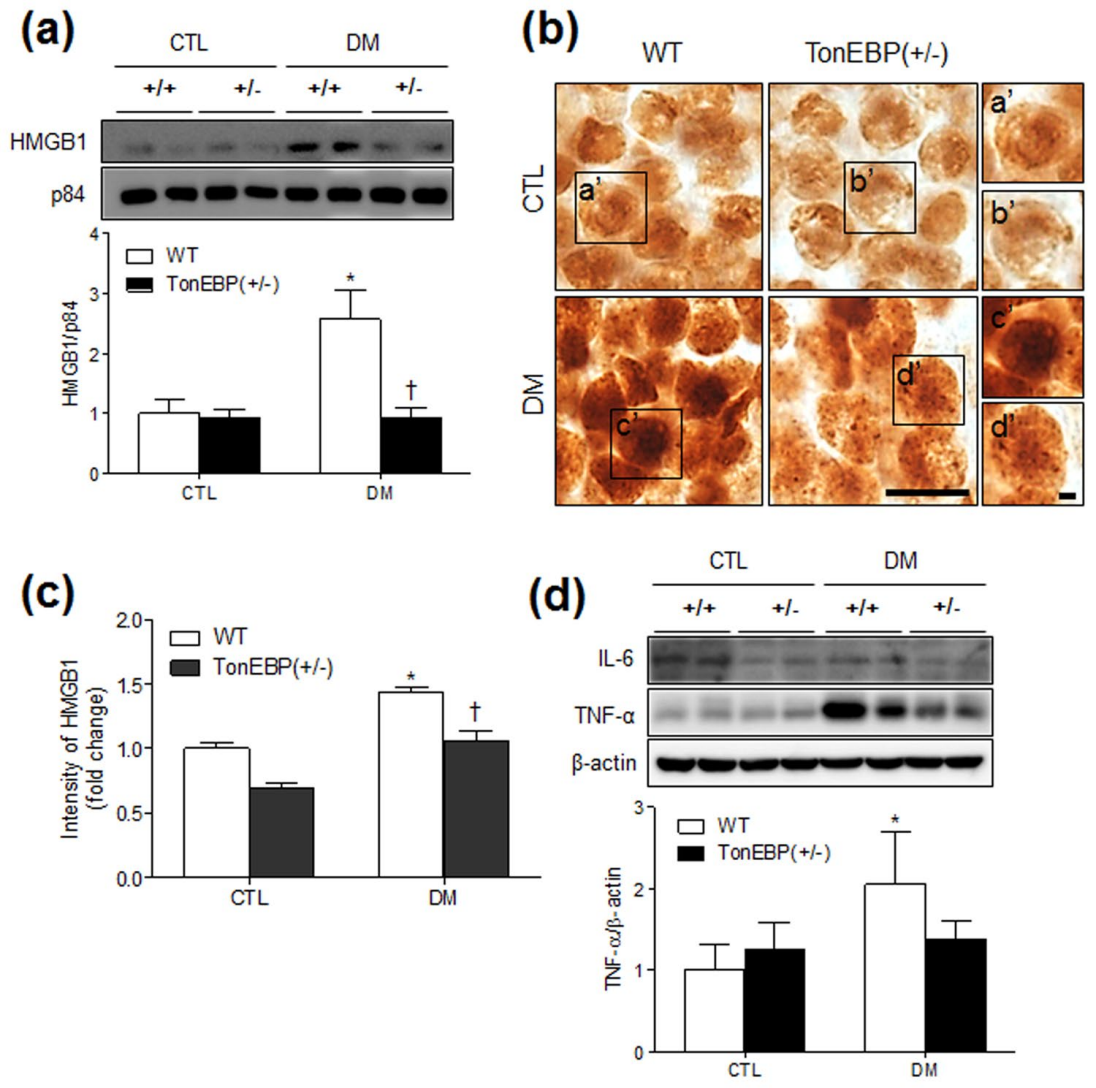
Effects of TonEBP haploinsufficiency on HMGB1, IL-6, and TNF-α expression in the hippocampus of HFD/STZ-treated mice. (**a**) Western blots and protein quantification of nuclear HMGB1 in the hippocampus. (**b**) Representative micrographs of HMGB1 immunoreactivity in the hippocampus. The boxed areas of a’, b’, c’, and d’ are magnified on the right. Scale bar = 25 μm (inset, 5 μm). (**c**) The intensity of HMGB1 immunoreactivity in the hippocampus was measured and presented as the fold change. (**d**) Western blots and protein quantification of hippocampal IL-6 and TNF-α. The mean values were obtained from three separate experiments (n = 6 mice per group). Cropped blots are displayed here and full-length blots are included in the Supplementary Information. Data are presented as the mean ± SEM. *P < 0.05 *vs*. control (CTL) normal diet-fed mice; ^†^ *P* < 0.05 *vs*. DM WT mice.

Using western blot and immunohistochemical analyses, we next confirmed that DM-induced HMGB1 expression is also reduced in the hippocampi TonEBP^+/−^ mice (Fig. 5). Whereas nuclear protein levels of HMGB1 were increased in WT mice with DM compared with those in their normal diet-fed counterparts, this increase was abolished in TonEBP^+/−^ mice (Fig. 5a). Accordingly, immunoreactivity staining showed that HFD/STZ-induced HMGB1 expression was lower in TonEBP^+/−^ hippocampal neurons than in WT mice (Fig. 5b,c). We next investigated HFD/STZ-induced proinflammatory cytokines in the hippocampus of WT and TonEBP^+/−^ mice (Fig. 5d). Although there was no change in hippocampal IL-6 protein expression between CTL and DM mice, HFD/STZ-treated WT mice exhibited increased hippocampal TNF-α expression compared with normal diet-fed WT mice. However, hippocampal TNF-α expression by haploinsufficiency of TonEBP was not significantly reduced in diabetic TonEBP^+/−^ mice. In addition, we detected HMGB1 expression in the nuclei of GFAP-positive astrocytes in the hippocampus (Supplementary Fig. 7). However, we did not observe a change in GFAP expression with HFD/STZ treatment. In accordance with hepatic findings, there was also no significant effect of TonEBP haploinsufficiency on NF-κB p65 expression with DM (Supplementary Fig. 4b).

### HFD/STZ-induced microglial activation is reduced in the hippocampi of TonEBP^+/−^ mice

**Figure 6:**
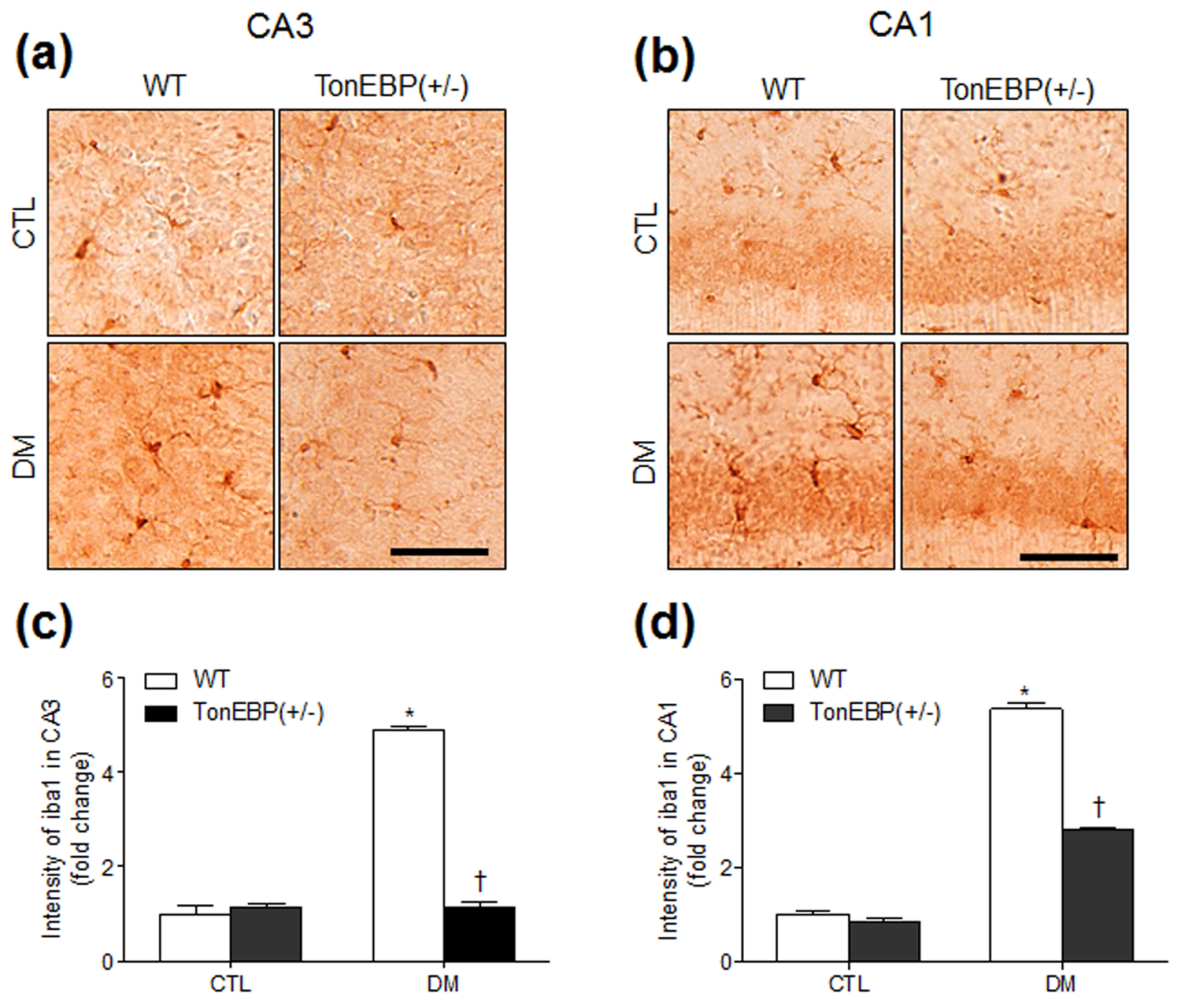
Effects of TonEBP haploinsufficiency on iba1 expression in the hippocampus of HFD/STZ-treated mice. Representative micrographs of iba1 immunoreactivity in the hippocampal CA3 (**a**) and CA1 (**b**) regions. The intensity of iba1 immunoreactivity in the hippocampal CA3 (**c**) and CA1 (**d**) regions was measured and presented as the fold change. Data are presented as the mean ± SEM. *P < 0.05 *vs*. control (CTL) normal diet-fed mice; ^†^ *P* < 0.05 *vs*. DM WT mice. Scale bar = 50 μm.

A recent study reported that TonEBP expression in microglia is upregulated in neuroinflammatory diseases^24^. Similarly, we also observed that TonEBP was detected in iba1-positive microglia in the hippocampus (Supplementary Fig. 8). The iba1 immunoreactivity was intense in CA1 and CA3 regions of HFD/STZ-treated WT mice but was comparatively dramatically lower in TonEBP^+/−^ mice with DM (Fig. 6a,b). We confirmed semiquantitatively that iba1 expression, which is upregulated upon microglial activation, was increased after HFD/STZ treatment (Fig. 6c,d).

## Discussion

Studies suggest that TonEBP upregulation is associated with many inflammatory diseases, including rheumatoid arthritis, atherosclerosis, seizures, lipopolysaccharide-injected brain injury, and diabetes^18, 24–26^. In this study, we demonstrate the role of TonEBP in DM-induced hepatic steatosis and neuroinflammation. We found elevated nuclear expression of TonEBP in diabetic livers and colocalization with HMGB1 in hippocampal neurons. We also found that TonEBP^+/−^ mice had reduced levels of hepatic lipogenic factors, HMGB1, IL-6, and TNF-α expression, as well as reduced macrophage infiltration and proinflammatory cytokines in adipose tissues of HFD/STZ-induced diabetes. We showed a significant reduction in iba1 expression in the hippocampi of diabetic mice with TonEBP haploinsufficiency. These findings indicate that TonEBP plays a critical role in the pathology of diabetes and that its inhibition significantly reduces HMGB1-mediated inflammation.

Obesity-induced chronic inflammation causes insulin resistance and leads to diabetes through a complex series of pathophysiological events characterized by hyperlipidemia, hyperglycemia, hepatic steatosis, systemic inflammation, and cognitive dysfunction. We used a mouse model mimicking human T2 diabetes, and characterized by hyperglycemia and abnormal insulin secretion caused by impaired β-cell function and insulin resistance^27–30^. In our studies, we also showed that chronic HFD-induced hyperinsulinemia was reduced by a single STZ injection and this led to a reduction of insulin levels.

In this model, mice were fed an HFD for 20 weeks to induce chronic low-grade inflammation and were then injected with STZ for high-glucose-induced hyperosmolarity. We showed that HFD/STZ-treated WT mice have higher fat mass, body weight, and serum glucose and cholesterol levels than control mice. Interestingly, there were significant reductions of fat mass and body weight in diabetic TonEBP^+/−^ mice compared with those of WT mice, although there was no difference in food intake between the groups. These findings suggest that TonEBP is an important regulator in adipogenesis.

Diabetes and insulin resistance are associated with nonalcoholic fatty liver disease^31^, and the livers of obese mice overexpress hepatic SREBP-1 and LXR through increased transcriptional regulation of TG synthesis^19, 32^. In addition, PPARα is mainly present in the liver and its activation results in *de novo* hepatic lipogenesis^33^. In this study, we found that HFD/STZ-induced hepatic steatosis was significantly reduced by TonEBP haploinsufficiency and was associated with lower levels of these factors. Our findings are consistent with the fact that hepatic SREBP-1, LXR, and PPARα expressions are increased in obese db/db mice and reversed by caloric restriction^34^. However, the suppression of DM-induced increases by TonEBP haploinsufficiency contradicts the effect of TonEBP on the adipogenesis in 3T3-L1 cells^26^. This may be due to the difference between *in vitro* and *in vivo* functions with or without low-grade inflammation (induced by a chronic HFD) and hyperglycemia (induced by STZ). Thus, these data indicate that the induction of lipogenic genes in response to insulin and glucose is under the control of TonEBP. In this study, we found that increased TonEBP expression in HFD/STZ-induced hepatic steatosis is suppressed by TonEBP haploinsufficiency.

A recent report showed that the progression of nonalcoholic steatohepatitis was reduced by inhibiting HMGB1–NF-κB translocation^35^. We found that haploinsufficiency of TonEBP suppressed the HFD/STZ-induced upregulation of HMGB1 in hepatic nuclei and attenuated hepatic steatosis. Furthermore, TonEBP haploinsufficiency abolished the increase in HMGB1 in the hippocampus induced by HFD/STZ treatment. These findings are consistent with the significant increase in HMGB1 observed in the spinal dorsal horns of db/db mice^36^, where the blockade of HMGB1 reduced diabetic pain by decreasing the expression of inflammatory mediators. Treatment with anti-HMGB1 neutralizing antibodies also reversed neuroinflammation and brain damage in diabetic rats with cerebral ischemia^37^. These data suggest that genetic haploinsufficiency of TonEBP is protective against hepatic steatosis and neuroinflammation induced by HMGB1 and TNF-α upregulation. In addition, we found that HFD/STZ-induced infiltration of macrophages in adipose tissue was reduced by TonEBP haploinsufficiency. Indeed, TonEBP haploinsufficiency was shown to significantly reduce atherosclerotic lesion formation and macrophage migration in an *in vivo* model of atherosclerosis^25^. Consistent with the reduction of IL-6 mRNA in macrophages lacking TonEBP^17^, TonEBP haploinsufficiency reduced the expression of IL-6 proteins in livers and adipose tissues of mice with DM.

Recent reports have demonstrated that the expression of TonEBP in neurons increases with acute and prolonged systemic hypertonicity^38, 39^. In this view, we hypothesized that TonEBP regulates osmoprotective neuroinflammation in the diabetic brain. Our findings support this hypothesis, showing that the reduction of TonEBP in mice with haploinsufficiency reduces HFD/STZ-induced HMGB1 expression. We also observed remarkable microglial activation in the hippocampi of HFD/STZ-treated mice, which was attenuated in TonEBP^+/−^ mice. Our findings are consistent with a recent report showing that TonEBP is induced in microglia in a rat with transient middle cerebral artery occlusion and in cultured cells treated with lipopolysaccharide^24^. Interestingly, inhibiting TonEBP significantly protected against microglial activation.

Our study consistently supports the notion that TonEBP haploinsufficiency suppresses neuroinflammation in the diabetic brain and reduces steatosis in the liver, suggesting that HFD/STZ-induced TonEBP may be a detrimental factor eliciting chronic inflammation. TonEBP functions as a transcriptional activator by binding to osmotic response elements of osmoprotective genes^13^ and interacting with the NF-κB p65 subunit^40^. In this view, we believe that TonEBP haploinsufficiency decreased HMGB1–NF-κB-mediated transcriptional activity and attenuated the severity of neuroinflammation. The results from this study suggest that TonEBP haploinsufficiency may have an anti-inflammatory effect in HFD/STZ-induced diabetes by reducing HMGB1-mediated inflammation.

## Materials and Methods

### Animals

Female and male TonEBP heterozygote (+/−) mice (6 weeks old) were received from Dr. Kwon (Ulsan National Institute of Science and Technology). The TonEBP^+/−^ mice were crossed to obtain TonEBP^+/−^ mice and their WT (TonEBP^+/+^) littermates. Animal experiments were performed in accordance with the National Institutes of Health Guide for the Care and Use of Laboratory Animals. The University Animal Care Committee for Animal Research of GNU approved the study protocol (GNU-150116-M0002). Mice were housed with an alternating 12-h light/dark cycle with food and water available. The diabetic mice were given an HFD (60% kcal fat; Research Diets, New Brunswick, NJ, USA) for 16 weeks. After that, mice received a single dose (100 mg/kg) of STZ (Sigma-Aldrich, St Louis, MO, USA) dissolved in 0.05 M sodium citrate buffer (pH 4.5) via intraperitoneal injection and followed by continued HFD feeding for an additional 4 weeks. WT mice were given a normal diet and injected with the sodium citrate buffer vehicle. Mice (*n* = 10 mice per group) were weighed and fasting blood glucose was measured monthly using an Accu-Chek glucometer (Roche Diagnostics GmbH, Mannheim, Germany). In addition, food intake (Supplementary Fig. 1) was measured two times each month before the animals were sacrificed.

### GTT and ITT

GTT and ITT were performed as previously described^38^. Briefly, d-glucose (2 g/kg; Sigma-Aldrich) or insulin (0.75 U/kg; Humulin-R, Eli Lilly, Indianapolis, IN, USA) was injected, and blood glucose was measured before and after the injection using an Accu-Chek glucometer (Roche Diagnostics GmbH).

### Measurement of metabolic parameters

After overnight fasting, mice (*n* = 10 mice per group) were deeply anesthetized with zoletil (5 mg/kg; Virbac Laboratories, Carros, France). The resultant serum samples were analyzed for serum glucose and aspartate aminotransferase (AST), alanine aminotransferase (ALT), and total cholesterol levels using enzymatic colorimetric assays from Green Cross Reference Laboratory (Yongin-si, South Korea). Mouse insulin kits (Shibayagi Co., Gunma, Japan) were used to measure insulin levels in serum (*n* = 10 mice per group). TG concentrations from frozen livers (*n* = 7–8 mice per group) were determined using a colorimetric assay kit from Cayman Chemical Company (Ann Arbor, MI, USA). The HOMA-IR was calculated from the fasting serum glucose (mg/dL) × fasting plasma insulin (mU/mL) divided by 405.

### Tissue analyses and histological staining

Mice (*n* = 4 mice per group) were perfused with 4% formaldehyde solution in 0.1 M phosphate-buffered saline (PBS) through the left ventricle after anesthesia by zoletil (5 mg/kg). After postfixation for 6 h, the brains and livers were sequentially immersed in 0.1 M PBS containing 15% sucrose and then in 30% sucrose at 4 °C until they were completely submerged. Frozen brains were cut into coronal sections (40 μm). To determine the intracellular lipid droplets in hepatic steatosis, frozen liver sections (10 μm) were stained with Nile red (Sigma, St. Louis, MO, USA) for 10 min. The livers were processed for paraffin embedding, and were then sectioned (5 μm) and stained with H&E. Images of the sections were captured using a BX51 light microscope (Olympus, Tokyo, Japan).

### Western blot analysis

After anesthesia by zoletil (5 mg/kg), hippocampi, liver, epididymal fat pads, and pancreas from mice (*n* = 6 mice per group) were quickly removed and frozen. Whole cellular extracts and nuclear fractions were prepared as previously described^34^. The nuclear fraction was separated by using a Nuclear and Cytoplasmic Extraction Kit (Pierce, Rockford, IL, USA) according to the manufacturer’s protocol. Western blot analyses were performed using standard methods. Frozen tissues were homogenized in lysis buffer for protein extraction. Proteins were immunoblotted with primary antibodies (Supplementary Table 1). The membranes were visualized using an enhanced chemiluminescence substrate (Pierce). The band densitometry was performed using a Multi-Gauge V 3.0 image analysis program (Fujifilm, Tokyo, Japan). To normalize the protein levels, p84, β-actin, or α-tubulin was used as an internal control.

### Immunofluorescence

Free-floating brain and deparaffinized pancreatic sections were incubated with primary antibodies at 4 °C for overnight (Supplementary Table 1). After washing three times with 0.1 M PBS, sections were incubated with Alexa Fluor 488-, 594-, and 790-conjugated donkey secondary antibodies (Invitrogen, Carlsbad, USA). Nuclei were stained with DAPI (1:10,000; Invitrogen). Fluorescence images of the sections were captured using a BX51-DSU microscope (Olympus).

### Immunohistochemistry

Free-floating brain, deparaffinized liver, adipose tissue, and pancreatic sections were incubated with primary antibodies that were diluted in blocking solution overnight at 4 °C (Supplementary Table 1). After three washes with 0.1 M PBS, sections were incubated for 1 h at room temperature with biotinylated secondary antibody. After washing, sections were incubated in an avidin-biotin-peroxidase complex solution (Vector Laboratories, CA, USA) and developed with diaminobenzidine (Vector Labs) containing 0.025% H_2_O_2_. Sections were then dehydrated through graded alcohol solutions, cleared in xylene, and mounted under a coverslip with Permount (Sigma-Aldrich, St. Louis, MO, USA). Image of the stained sections were captured using a BX51 light microscope (Olympus). Immunohistochemical intensity data for insulin, C-peptide, TonEBP, HMGB1, and iba-1 were obtained from selected images using i-Solution (IMT i-Solution Inc., Vancouver, BC, Canada). Three fields (200 × 200 µm^2^) were randomly selected on each section from two continuous sections (n = 4 mice per group). Intensity measurements are represented as the percentage of the mean number of pixels versus the corresponding value at which the pixel of the respective intensity was present.

### Statistical analysis

Group differences were determined by one-way analyses of variance (ANOVA) followed by Bonferroni post-hoc analyses using PRISM (GraphPad Software Inc., San Diego, CA, USA). Two-way ANOVA with repeated measures analyses of variances was used for weight gain, fasting blood glucose, ITT, and GTT. Values are expressed as the means ± standard errors of the mean (SEM). A *P* value < 0.05 was considered as statistically significant.

## Electronic supplementary material

Supplementary Information
