# Supplementary material for: TonEBP/NFAT5 haploinsufficiency attenuates hippocampal inflammation in high-fat diet/streptozotocin-induced diabetic mice
Source: Sci Rep. 2017 Aug 10;7:7837. doi: 10.1038/s41598-017-08319-w (PMC5552681; doi:10.1038/s41598-017-08319-w)
Supplement: Supplementary file 1 — Supplementary Information [file 41598_2017_8319_MOESM1_ESM.pdf]

TonEBP/NFAT5 haploinsufficiency attenuates hippocampal inflammation in high-fat diet/streptozotocin-induced diabetic mice

Jong Youl Lee[1,2], Eun Ae Jeong[1,2], Kyung Eun Kim[1,2], Chin-ok Yi[1,2], Zhen Jin[1,2], Jung Eun Lee[3], Dong Hoon Lee[1], Hyun Joon Kim[1,2], Sang Soo Kang[1], Gyeong Jae Cho[1], Wan Sung Choi[1,2], Soo Youn Choi[4], H. Moo Kwon[4], Gu Seob Roh[1,2]*

Supplementary Table 1. List of primary antibodies

Antibody	Company	Catalog No.	Dilution	Applications	Source
Insulin	Abcam	Ab7842	1:100	IF	Guinea Pig
NF-κB p65	Cell signaling	#6956	1:1000	WB	Mouse
p84	Abcam	Ab487	1:1000	WB	Mouse
LXRβ	R&D	K8917	1:1000	WB	Mouse
SREBP-1	BD	557036	1:1000	WB	Mouse
PPARα	Abcam	Ab8934	1:1000	WB	Rabbit
TonEBP	Dr. Kwon		1:3000,1:200	WB,IHC,IF	Rabbit
TonEBP	Santa cruz	Sc-5501	1:50	IF	Goat
GFAP	Santa cruz	Sc-6171	1:500	IF	Goat
IL-6	Santa cruz	Sc-1265	1:1000	WB	Goat
TNF-α	Cell signaling	#3707	1:1000	WB	Rabbit
C-peptide	Abcam	Ab14181	1:1000	IHC	Rabbit
NeuN	Millipore	MAB377	1:500	IF	Mouse
HMGB1	Abcam	Ab18256	1:1000,1:100	WB,IHC,IF	Rabbit
Iba1	Wako	016-20001	1:150	IHC	Rabbit
CD68	Santa cruz	Sc-9139	1:75	IHC	Rabbit
β-actin	Sigma	A5441	1:30000	WB	Mouse
α-tubulin	Sigma	T5168	1:30000	WB	Mouse

WB, western blot; IF, immunofluorescence; IHC, immunohistochemistry

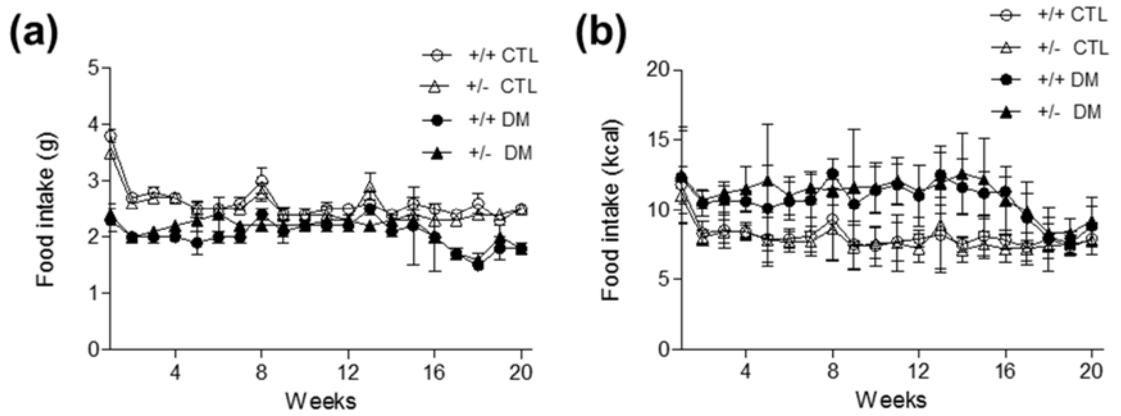

Supplementary Figure 1. Food intake and total calorie intake of control or HFD/STZ-treated WT and TonEBP$^{+/-}$ mice. Food intake (g) **(a)** and total calorie intake calculated from the amount of food intake (kcal) (**b**) by each group of mice; control diet = 3.1 kcal/g, HFD = 5.24 kcal/g (60 kcal% fat).

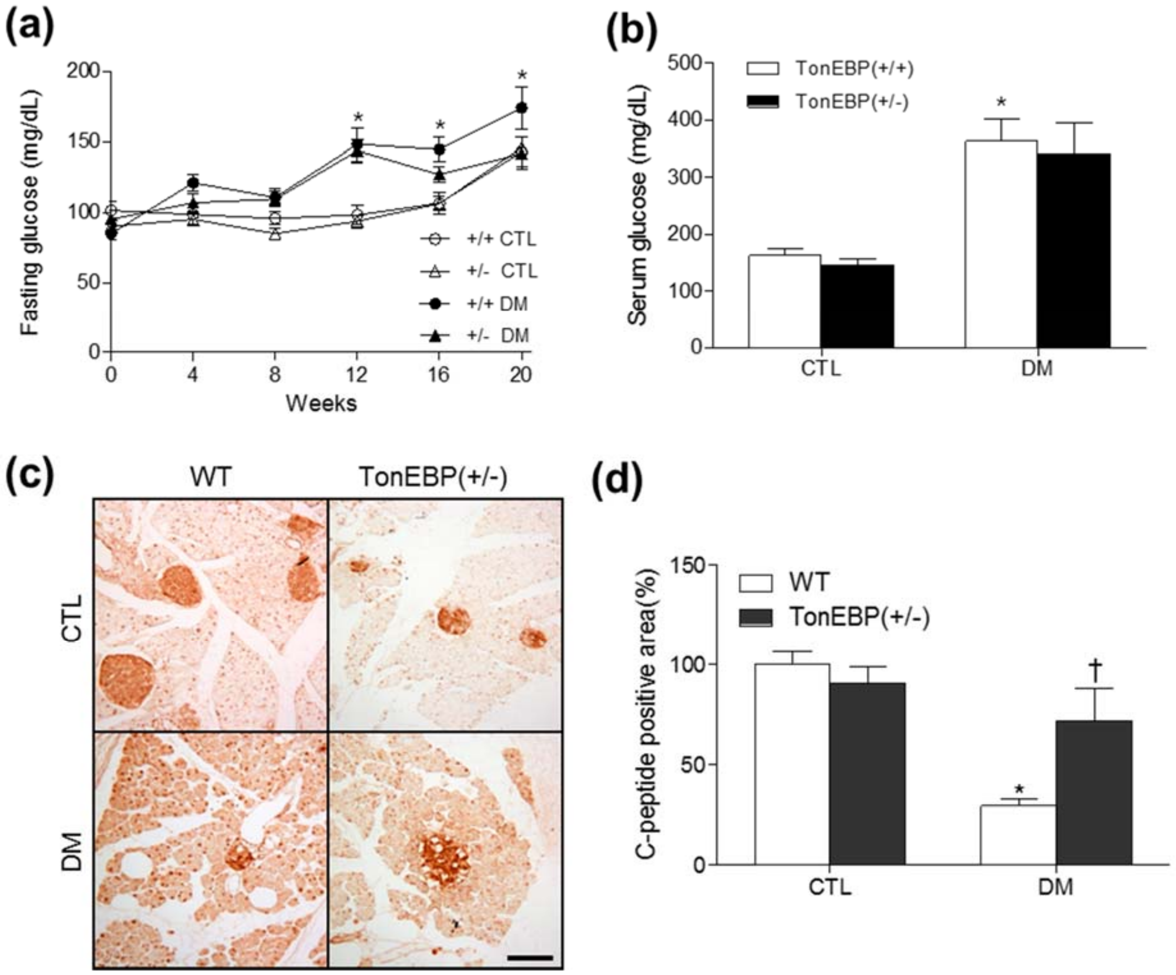

Supplementary Figure 2. Effects of TonEBP haploinsufficiency on glucose levels and pancreatic C-peptide expression in HFD/STZ-treated mice. Fasting blood glucose **(a)** for 20 weeks and serum glucose **(b)** in control or HFD/STZ-treated WT and $TonEBP^{+/-}$ mice. **(c)** Representative images showing immunofluorescence of C-peptide in pancreatic sections. **(d)** Percentage areas of C-peptide-positive cells. Data are shown as the mean ± SEM. **P* < 0.05 *vs*. control (CTL) normal diet-fed mice; $^{\dagger}P < 0.05$ *vs*. DM WT mice. Scale bar = 100 μm.

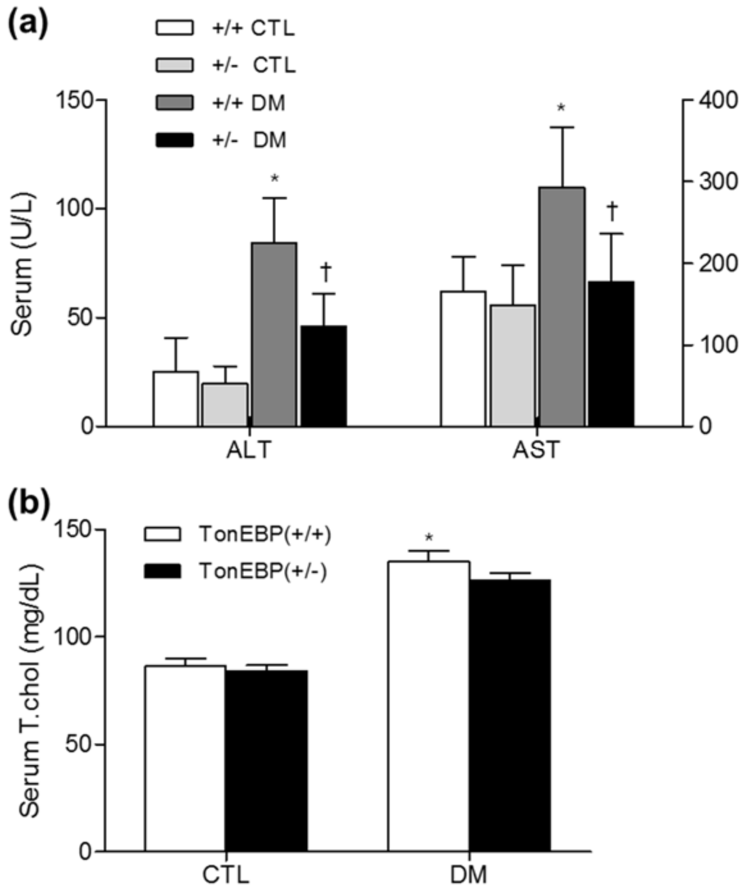

Supplementary Figure 3. Effects of HFD/STZ treatment on hepatic enzymes and serum total cholesterol in WT and TonEBP$^{+/-}$ mice. Serum hepatic alanine aminotransferase (ALT), aspartate aminotransferase (AST) **(a)**, and total cholesterol **(b)** levels were determined by enzymatic colorimetric assays. Data (n = 10 mice per group) are presented as the mean ± SEM. **P* < 0.05 *vs*. control (CTL) normal diet-fed mice; $^{\dagger}P$ < 0.05 *vs*. DM WT mice.

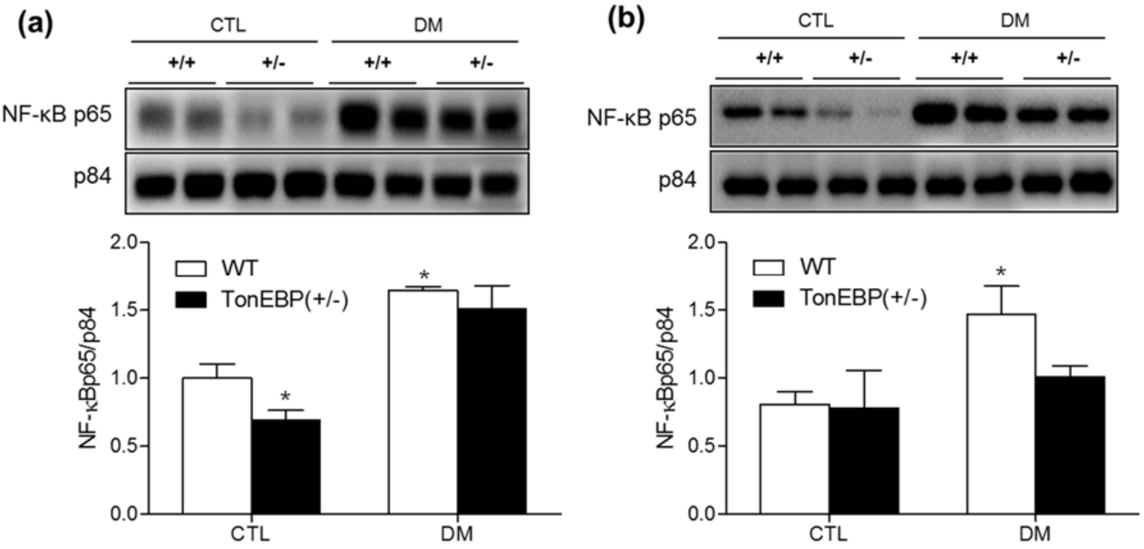

Supplementary Figure 4. Hepatic and Hippocampal NF-κBp65 expression in HFD/STZ-treated TonEBP$^{+/-}$ mice. Western blots and protein quantification of nuclear TonEBP in the liver (**a**) and hippocampus (**b**). Data are presented as the mean ± SEM from three separate experiments (n =6 mice per group). *$P < 0.05$ *vs*. control (CTL) normal diet-fed mice.

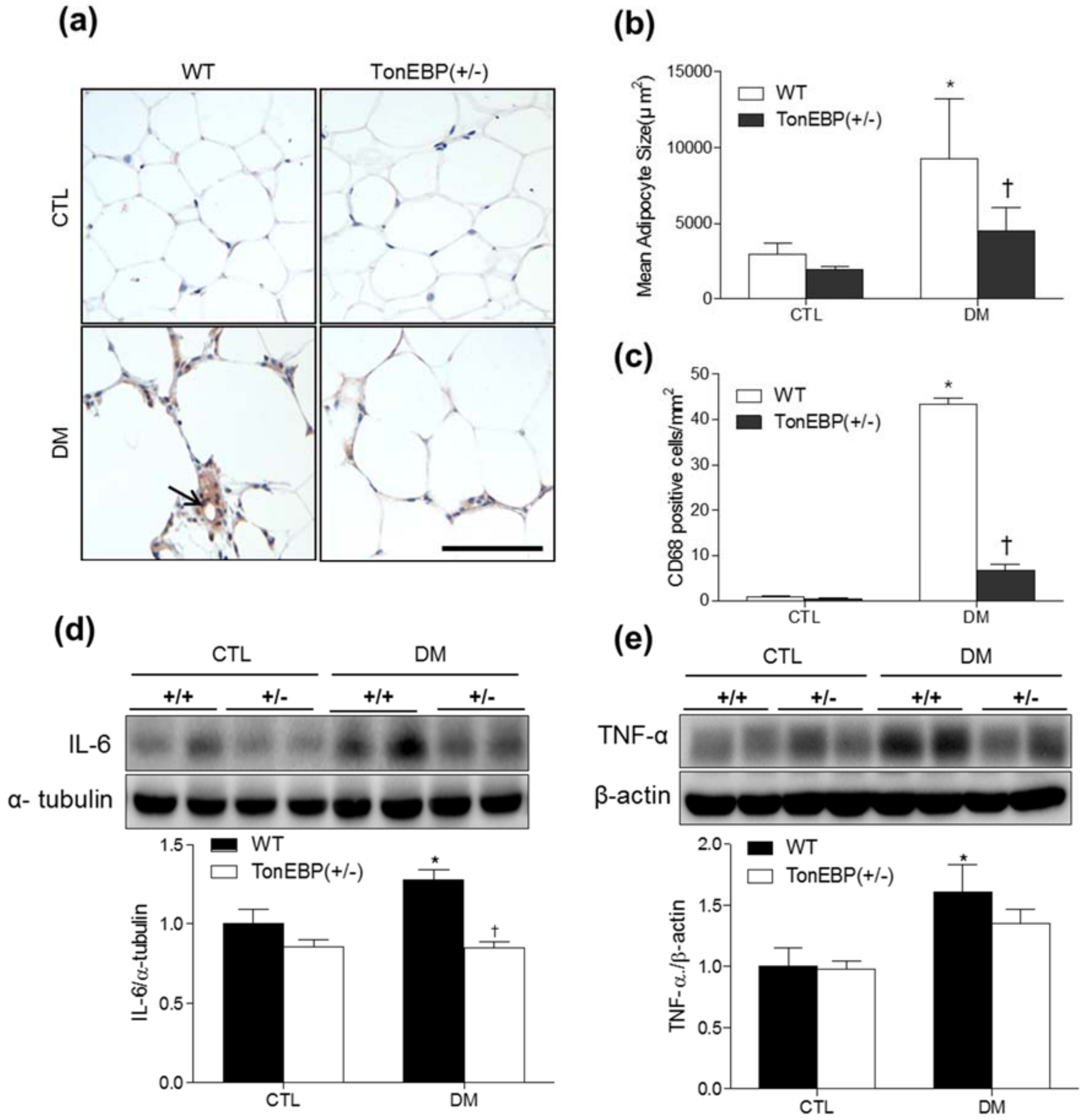

Supplementary Figure 5. Effects of TonEBP haploinsufficiency on macrophage infiltration and proinflammatory cytokine expression in adipose tissues of HFD/STZ-treated mice. (a) Representative micrographs of CD68-immunostained liver sections; scale bar = 50 μm. Arrow indicates a macrophage. Mean adipose size **(b)** and number **(c)** of CD68-positive cells. Western blotting and quantification of IL-6 **(d)** and TNF-α **(e)** in epididymal fat pad tissues. Data are presented as the mean ± SEM from three separate experiments (n = 6 mice per group). *$P < 0.05$ *vs*. control (CTL) normal diet-fed mice; †$P < 0.05$ *vs*. DM WT mice.

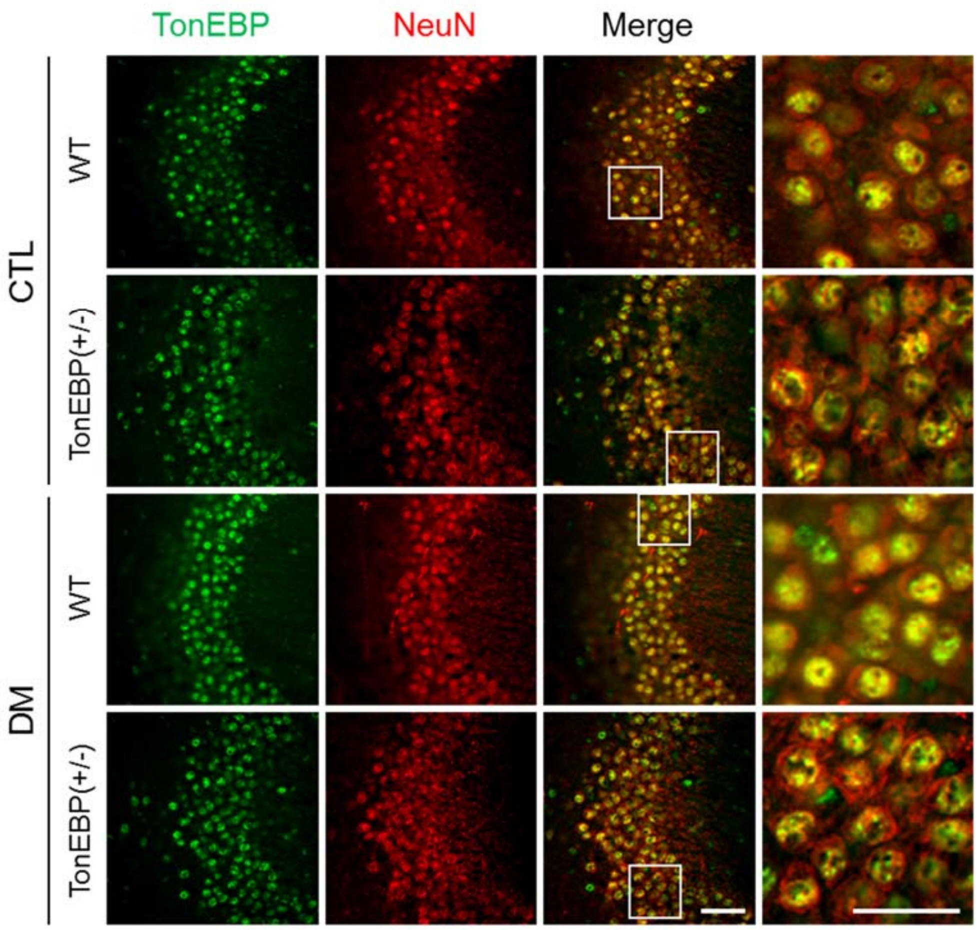

Supplementary Figure 6. Hippocampal TonEBP expression in HFD/STZ-treated TonEBP$^{+/-}$ mice. Representative micrographs of TonEBP (green) and NeuN (red)-immunostained hippocampal CA3 sections; scale bar = 50 μm (inset, 25 μm).

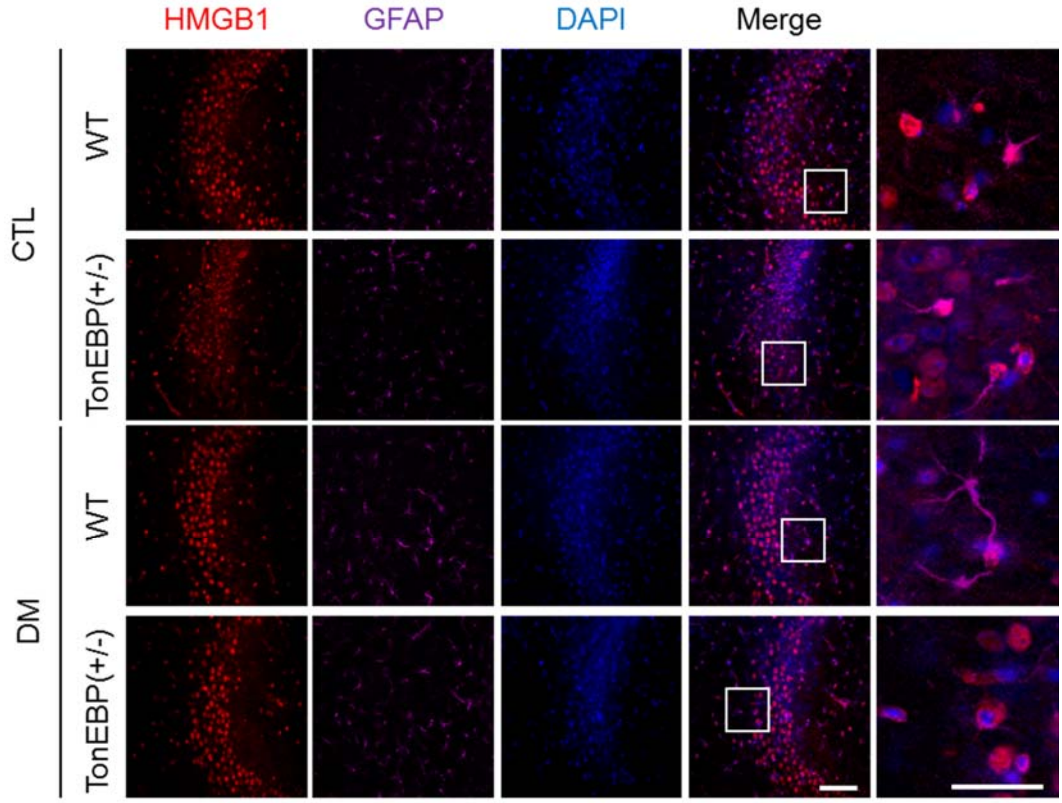

Supplementary Figure 7. Hippocampal HMGB1 and GFAP expression in HFD/STZ-treated TonEBP$^{+/-}$ mice. Representative micrographs of HMGB1 (red) and GFAP (purple)-immunostained hippocampal CA3 sections from control (CTL) mice and HFD/STZ-treated (DM) mice; scale bar = 50 μm (inset, 25 μm).

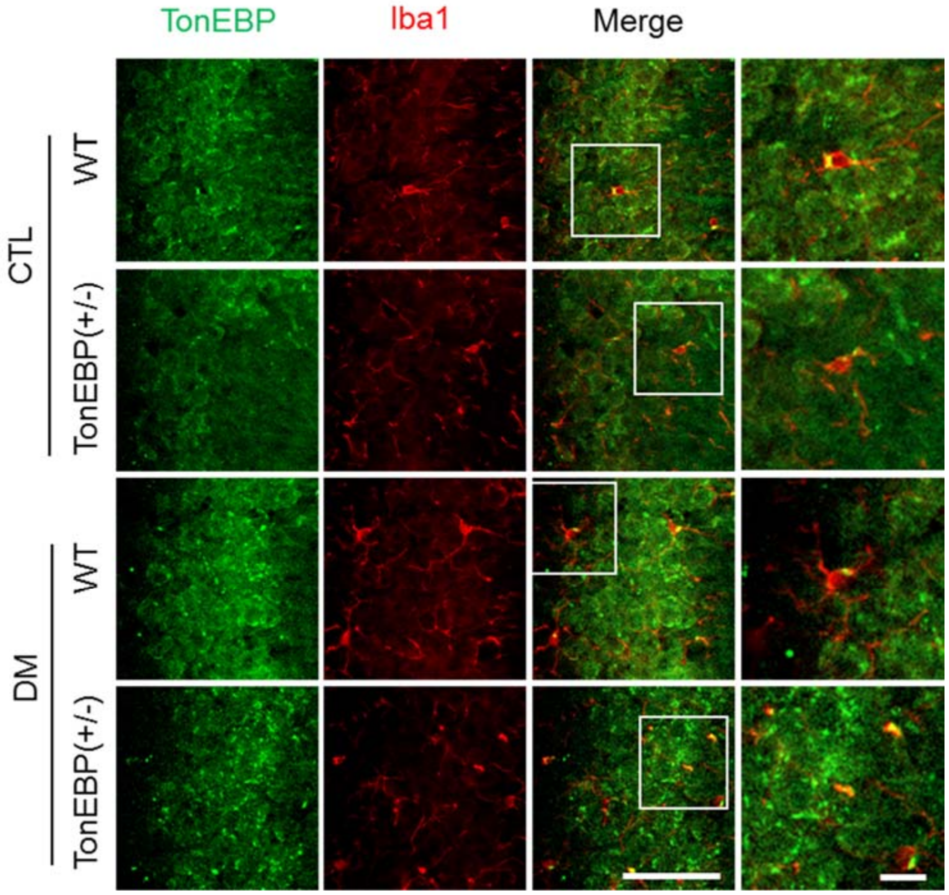

Supplementary Figure 8. Hippocampal iba1 expression in HFD/STZ-treated TonEBP$^{+/-}$ mice. Representative micrographs of TonEBP (green) and iba1 (red)-immunostained hippocampal CA3 sections from control (CTL) mice and HFD/STZ-treated (DM) mice; scale bar = 50 μm (inset, 25 μm).

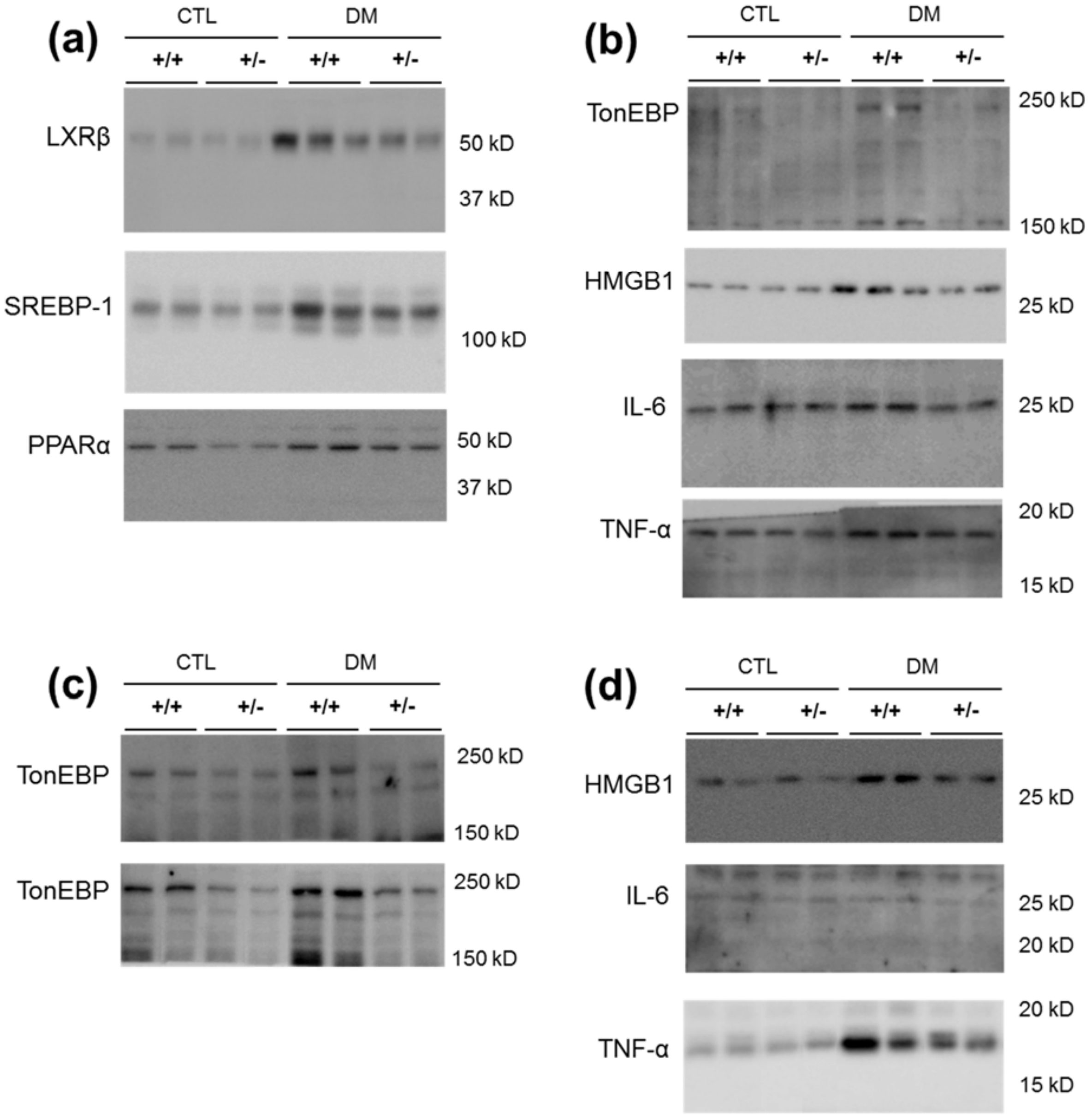

Supplementary Figure 9. (a) Full-length blots and multiple exposures of Figure 2d-f. **(b)** Full-length blots and multiple exposures of Figure 3b-f. **(c)** Full-length blots and multiple exposures of Figure 4a,b. **(d)** Full-length blots and multiple exposures of Figure 5a,d.
